# Microdomains form on the luminal face of neuronal extracellular vesicle membranes

**DOI:** 10.1038/s41598-020-68436-x

**Published:** 2020-07-20

**Authors:** Doreen Matthies, Nathanael Y. J. Lee, Ian Gatera, H. Amalia Pasolli, Xiaowei Zhao, Hui Liu, Deepika Walpita, Zhe Liu, Zhiheng Yu, Maria S. Ioannou

**Affiliations:** 1 Janelia Research Campus, Howard Hughes Medical Institute, Ashburn, VA USA; 2 Department of Physiology, University of Alberta, Edmonton, AB Canada; 3 Neuroscience and Mental Health Institute, University of Alberta, Edmonton, AB Canada; 4 Group On the Molecular and Cell Biology of Lipids, University of Alberta, Edmonton, AB Canada

**Keywords:** Membrane trafficking, Cellular neuroscience, Cell biology, Neuroscience

## Abstract

Extracellular vesicles (EVs) are important mediators of cell-to-cell communication and have been implicated in several pathologies including those of the central nervous system. They are released by all cell types, including neurons, and are highly heterogenous in size and composition. Yet much remains unknown regarding the biophysical characteristics of different EVs. Here, using cryo-electron microscopy (cryoEM), we analyzed the size distribution and morphology of EVs released from primary cortical neurons. We discovered massive macromolecular clusters on the luminal face of EV membranes. These clusters are predominantly found on medium-sized vesicles, suggesting that they may be specific to microvesicles as opposed to exosomes. We propose that these clusters serve as microdomains for EV signaling and play an important role in EV physiology.

## Introduction

Extracellular vesicles (EVs) are membrane-bound vesicles that are released by a variety of cell types, including those of the nervous system^1^. They are essential components in mediating cell-to-cell communication and have been implicated in a variety of diseases^2–5^. While there has been growing interest in understanding EV biology, much remains unknown regarding the biophysical properties of EVs released from different cell types.

EVs are a heterogenous population of vesicles that can be classified into two main subtypes based on their biogenesis. The inward budding of endosomes forms multivesicular bodies that fuse with the plasma membrane to release exosomes roughly 30–100 nm in diameter^6^. Microvesicles form by the outward budding of the plasma membrane and are typically larger in size: from 100–1,000 nm^6^. Because exosomes and microvesicles have different molecular origins, they are expected to have distinct biochemical properties and likely distinct physiological functions. However, whether different EV subtypes (or EVs of different sizes) have different biophysical characteristics is poorly understood^7^.

The small size of EVs makes them challenging to study; they are 2–3 times smaller than the diffraction limit of light microscopy. Conventional transmission electron microscopy (TEM) has provided invaluable information in our current understanding of EVs. However, the process of dehydration, embedding, and staining used in conventional TEM can lead to structural artefacts in addition to offering limited resolution to resolve small structural details within EVs^8^. CryoEM preserves EV structure in its close-to-native hydrated state and provides high-resolution images, making it the ideal tool for characterizing biophysical properties of EVs^9–14^.

Previously, EVs released from cultured cell lines or blood serum have been studied using cryoEM, providing important information on their size and morphology^11,12^. The size and quantity of EVs released from neurons infected with Zika virus have also been described using cryoEM, revealing the role of EVs in viral transmission^13^. A detailed morphological characterization of EVs released from neurons in their native state is needed to gain insight into the role of EVs in the physiology of healthy neurons.

Here, we performed high-resolution cryoEM imaging of EVs released from primary cortical neurons. We found that neuron-derived EVs display a similar size distribution compared to non-neuronal cell types; however, irregularly shaped EVs (tubules or pleomorphic vesicles) were rarely observed^11,12^. Excitingly, we discovered dense macromolecular clustering on the luminal face of EV membranes. These clusters were observed in medium-sized EVs, suggesting that they may be a specific feature of microvesicles. We speculate that these macromolecular clusters serve as functional microdomains in EVs released from a variety of cell types and play an important role in EV physiology.

## Results

### Neurons release small- and medium-sized EVs

An important question in EV biology is whether EVs released by specific cell types vary in size or morphology^7^. EVs typically exhibit a range of sizes from 30–250 nm^11–13^ and various morphologies^11,14^. Since different EV concentration protocols can affect the mean size distribution of vesicles and, in some cases, induce morphological changes^15^, we first sought to characterize the size distribution of neuronal EVs concentrated using two different methods: differential ultracentrifugation (dUC) and ultrafiltration (UF).

**Figure 1:**
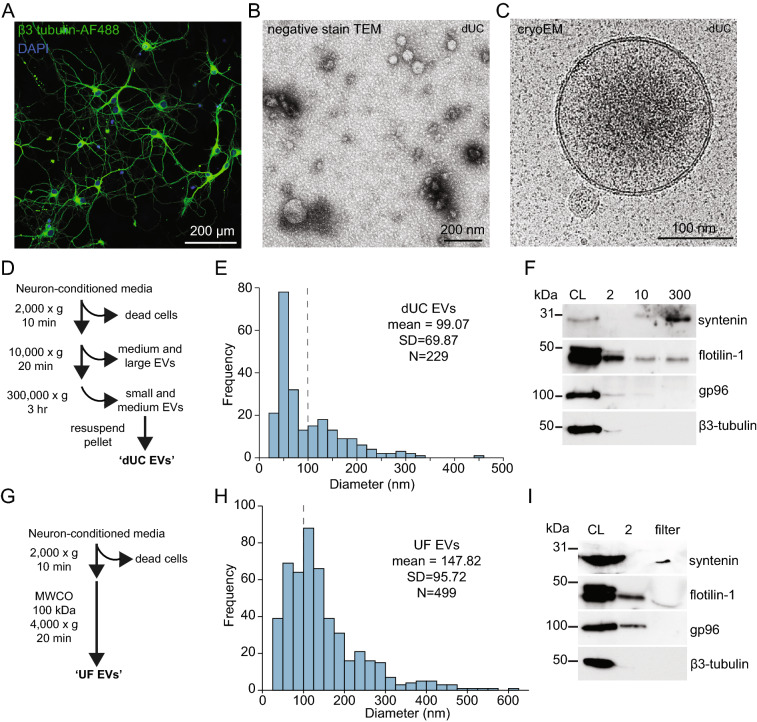
(**A**) Cortical neurons were fixed and immunostained for neuron-specific β3-tubulin (Tuj). (**B**) Transmission electron micrograph of EVs purified from neuron-conditioned media observed by negative staining. (**C**) Representative image of EVs imaged by cryoEM. (**D**) Schematic of procedures used to concentrate EVs by differential centrifugation (dUC). (**E**) Frequency distribution of dUC EV diameter determined by cryoEM. Dotted line separates small and medium-sized EVs. (**F**) Western blotting of centrifugation fractions prepared as in D. CL, cell lysate. Original unprocessed blots can be found in Supplementary Fig. S1. (**G**) Schematic of procedures used to concentrate EVs by ultrafiltration (UF). (**H**) Frequency distribution of UF EV diameter determined by cryoEM. Dotted line separates small and medium-sized EVs. (**I**) Western blotting of ultrafiltration fractions prepared as in G. CL, cell lysate. Original unprocessed blots can be found in Supplementary Fig. S1.

We grew primary cortical neurons from P0 rat pups for 10 days to allow neurons to differentiate and form functional connections^16–18^ (Fig. 1A). Cultured neurons are routinely grown in serum-free media thereby eliminating the risk of extracellular vesicles present in serum to influence subsequent analyses^16^. Neurons were grown in AraC to prevent growth of contaminating glial cells. Neuron-conditioned medium was collected and subjected to low-speed centrifugation (10,000×g for 20 min) to remove the cell debris and some large vesicles. EVs in the supernatant were then purified by ultra-high-speed centrifugation (300,000×g for 3 h) (Fig. 1D). This ultra-high speed allows a greater proportion of vesicles to be pelleted, as a considerable number of EVs remain in the supernatant following 100,000×g centrifugation^19,20^.

We verified the presence of vesicles by negative staining with TEM imaging (Fig. 1B). We next turned to cryoEM to obtain high-resolution images of EVs in their native hydrated state. Based on our cryoEM imaging, we found that neuronal EVs separated by dUC exhibited a broad range of sizes, from 25–450 nm in diameter (Fig. 1C–E). A bimodal distribution consisting of small (sEV; < 100 nm) and medium-sized (mEV; 100–500 nm) vesicles was observed (Fig. 1E) with a mean diameter of 99.07 nm.

Western blotting of dUC vesicles reveals an enrichment of the EV marker syntenin (Fig. 1F)^10,21,22^. Consistent with proteomics characterization of EVs^10,22^, flotillin-1 was present but not enriched in the dUC EVs (Fig. 1F). Neuron-specific β3-tubulin was not detected in our concentrated EVs, nor was the endoplasmic reticulum-resident marker gp96 (Fig. 1F)^10,22^. In addition to exosomes and microvesicles, unhealthy cells release large vesicles called apoptotic bodies^23^. The lack of gp96 detected suggests that apoptotic bodies are unlikely to be present in EVs concentrated by dUC^10,22,24^.

We also concentrated EVs using UF as a second approach (Fig. 1G). This approach does not subject vesicles to ultra-high g-force and eliminates the need to mechanically resuspend vesicles from a pellet^25,26^. Neuron-conditioned medium was collected and centrifuged at 2,000×g to remove dead cells. The supernatant was concentrated using a centrifugal filter unit with a 100 kDa molecular weight cut-off by centrifuging for 20 min at 4,000×g (Fig. 1G)^26^. Based on our cryoEM imaging, we found that neuronal EVs concentrated by UF were larger than those concentrated by dUC, with a mean diameter of 147.82 nm (Fig. 1H). This is likely due to the removal of many mEVs by the 10,000×g centrifugation step used when concentrating EVs by dUC (Fig. 1D,E). Like the dUC EVs, UF EVs contained syntenin and flotillin-1 but not the organelle marker gp96 by Western blot, indicating that apoptotic bodies are unlikely to be present in EVs concentrated by ultrafiltration (Fig. 1I).

As expected, our data shows that cortical neurons release a range of EV sizes. The difference in average EV diameter from 99 to 148 nm illustrates the ability of different concentration methods to selectively enrich different sized vesicles. Although the identity of a vesicle cannot be determined based on size alone, the distribution of EV sizes suggests that neurons may release exosomes and microvesicles.

### Macromolecular clustering occurs on luminal membranes of mEVs

**Figure 2:**
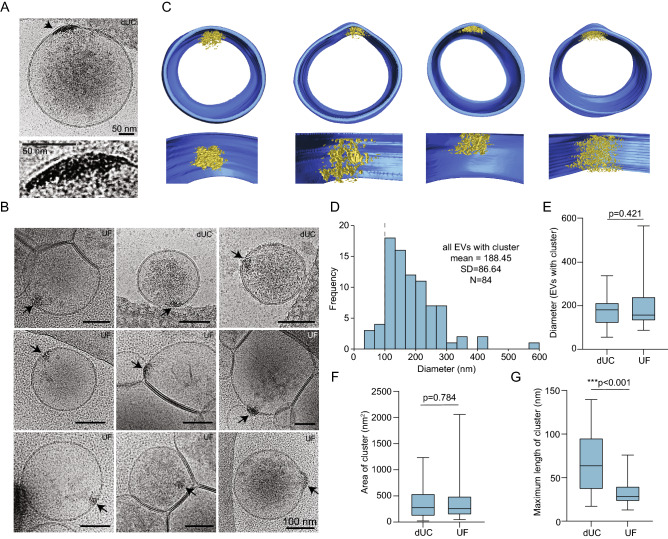
(**A**,**B**) CryoEM of EVs purified from neurons by dUC or UF. Black arrow shows electron-dense macromolecular clustering on membrane. The concentrated clustering region is magnified in the bottom image in A. (**C**) Three-dimensional organization of the macromolecular clusters (yellow) on the membranes (blue) of dUC EVs using cryo electron tomography (cryoET). Corresponds to Movie S1. (**D**) Frequency distribution of EVs containing clusters determined by cryoEM. Dotted line separates small and medium-sized EVs. Comparison of mean +/− SD by method of concentration: EV diameter (**E**), macromolecular cluster area (**F**) and maximum length of macromolecular cluster area (**G**). n = 21 for dUC and n = 63 for UF; Student’s *t*-test.

We next sought to characterize the biophysical properties of neuronal EVs by cryoEM. We discovered dense macromolecular clustering on the luminal face of EV membranes (Fig. 2A,B, black arrows). Reconstructed 3-dimensional tomograms confirm that these clusters are within vesicles as opposed to on the surface and are often present where the membrane bulged outward (Fig. 2C, cluster in yellow, Movie S1). Clusters were found predominantly in mEVs with an average vesicle size of 188.45 nm and were observed in 9.2% (21/229) of dUC EVs and 12.6% (63/499) of UF EVs (Fig. 2D). No difference in the size of EVs containing the clusters was observed between the two methods of EV concentration (Fig. 2E). The slightly higher abundance of clusters in UF EVs is consistent with the larger size distribution of vesicles by UF (Fig. 1). There was no difference in the mean 2-dimensional area of the clusters imaged from dUC EVs (403 nm^2^) and UF EVs (432 nm^2^) (Fig. 2F). The mean maximum length of the clusters was larger in dUC EVs (67 nm) compared to UF EVs (33 nm) (Fig. 2G).

**Figure 3:**
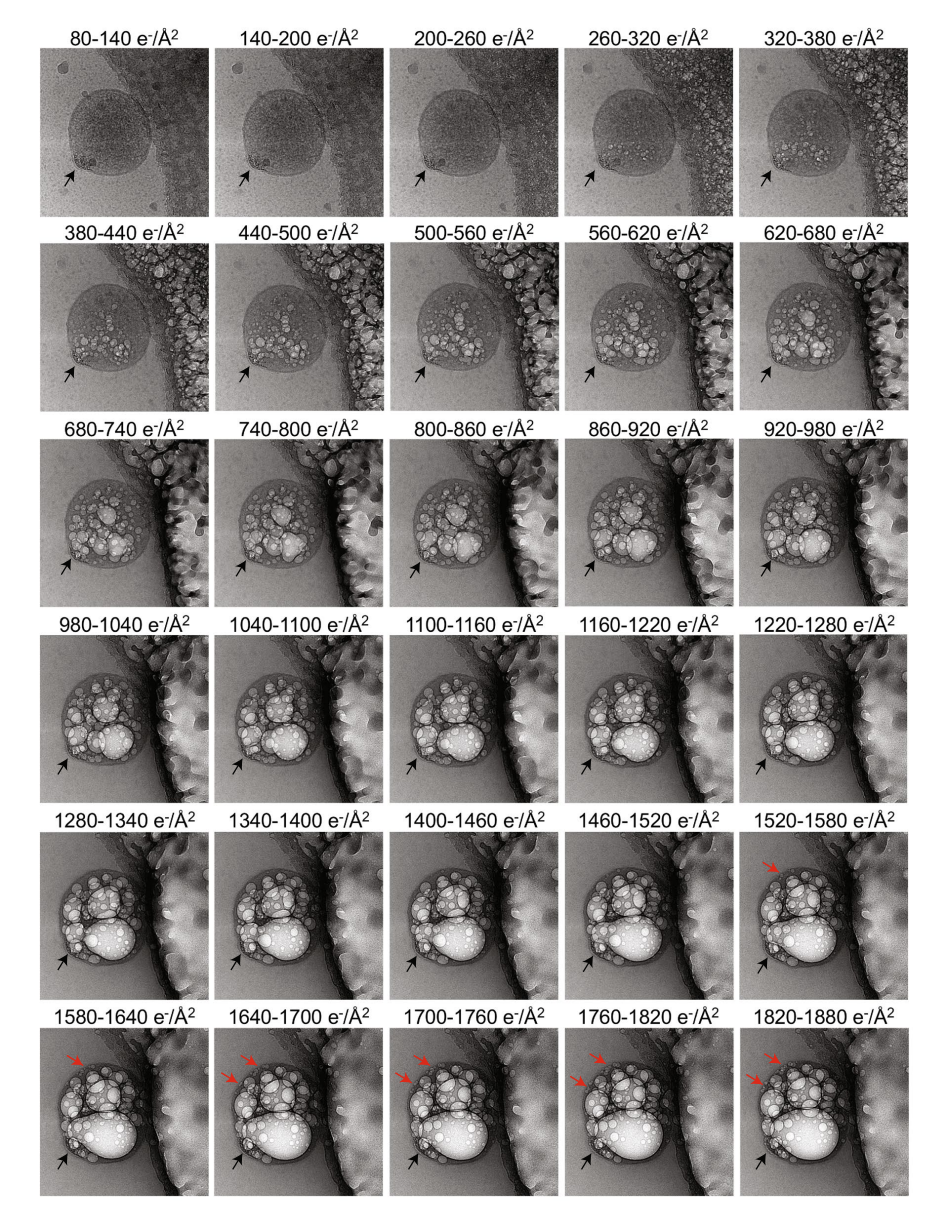
EV cluster resistant to radiation damage. dUC EV was exposed to 80 e^−^/Å^2^ during the acquisition of a tomogram. The same vesicle was imaged again multiple times with a dose of ~ 60 e^−^/Å^2^ for each additional image. At a total dose of > 260 e^−^/Å^2^, gas bubble formation due to radiation damage is visible in the vesicle lumen. The dense cluster (black arrow) is resistant to the long exposure, not showing local bubble formation. Red arrow indicates lipid bilayer damage.

Taking advantage of the fact that different macromolecules show varying susceptibility to radiation damage allowed us to probe the composition of these clusters^27^. Clusters were more resistant than the EV lumen to radiation damage as assessed by long electron exposure and gas/bubble formation (Fig. 3, black arrow). This suggests that the macromolecular environment within the cluster is distinct from the aqueous lumen. This also suggests that the clusters may be rich in phosphate, as phosphate-containing molecules are more resistant to radiation damage^27^. The clusters were more resistant to radiation damage than large regions of the phospholipid-rich plasma membrane (Fig. 3, red arrow). We speculate that these clusters are largely protein-based, may contain phosphate-rich molecules such as RNA, and likely form a distinct microdomain.

### Neuronal EVs are largely homogenous in morphology

**Figure 4:**
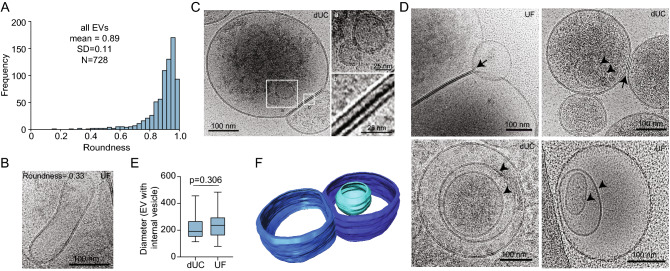
(**A**) CryoEM images of EVs were quantified for roundness. More round = 1, less round = 0. (**B**) Example EV that is elongated/oval. (**C**) EVs containing internal vesicles (magnified in a) and making membrane contacts with other EVs (magnified in b). (**D**) Example EVs containing internal vesicles (arrowheads) and contacting other EVs (arrows). (**E**) Comparison of EV diameter containing internal vesicles by method of concentration. Mean +/− SD. n = 21 for dUC and n = 63 for UF; Student’s *t*-test. (**F**) Three-dimensional reconstruction showing small vesicle (light blue) within larger vesicle (dark blue) and membrane-membrane contacts between two larger vesicles using cryo electron tomography (cryoET). Corresponds to Movie S2.

We next characterized the morphology of EVs released from primary neurons to identify any similarities or differences to EVs reported from non-neuronal cell types. EVs have a variety of reported morphologies, including round, tubular, and pleomorphic/irregular^11^. EVs released from neurons were predominantly round with 98.2% (715/728 EVs) having a roundness of greater than 0.5 (Fig. 4A–C). 2/229 (0.9%) of dUC EVs and 11/499 (2.2%) UF EVs displayed an oval morphology (roundness of less than 0.5) (Fig. 4A,B). We did not detect any coated vesicles or vesicles exhibiting electron-dense protrusions^11,28^. We detected a single vesicle (1/728 EVs) containing filamentous structures, suggesting that this is not common for EVs concentrated from neuron-conditioned medium by either method. This is consistent with undetectable levels of cellular markers gp96 and tubulin by Western blotting (Fig. 1F,I). Collectively, our imaging data suggest that EVs released from neurons are largely homogenous in morphology.

### Neuronal EVs contain internal vesicles

Similar to EVs from non-neuronal cells^11^, many neuronal EVs contained what appears to be one or more internal vesicles (Fig. 4C–D). Vesicles containing one or more internal vesicles were observed in 20/229 (8.7%) of dUC EVs and 48/499 (9.6%) of UF EVs. The mean diameter of EVs containing internal vesicles did not differ by method of concentration (Fig. 4E). Based on imaging of a single plane alone, it is not possible to tell whether the smaller vesicles are within the larger vesicle, or whether they are above or below the large vesicle. Using 3-dimensional cryo-electron tomography (cryoET), we confirmed that the small vesicles were indeed within the EV (Fig. 4F, Movie S2). Our data indicate that like other cell types, a subset of EVs concentrated from neuron-conditioned media contain internal vesicles. It remains unknown how these internal vesicles form, whether they exist in vivo, and what the physiological significance of these structures may be, if any.

### Neuronal EVs make membrane contact with other EVs

Finally, we noted that several neuronal EVs formed contacts with other EVs (Fig. 4C,D,F). This phenotype has previously been described as an artefact caused of high-speed centrifugation^15^. We observed EV-EV contacts more frequently when concentrated by UF (95/499 EVs or 19.0%) compared to those concentrated by dUC (16/229 or 7.0%). While our data does not preclude the possibility that EV-contacts are caused by the procedures used to concentrate them, it does reveal that EV contacts are not caused solely by high-speed centrifugation^15^.

## Discussion

Here, we used high-resolution cryoEM to image EVs released from cultured neurons in their native hydrated state. We found that neuronal EVs exhibit a broad range of sizes ranging from 25 to 600 nm. As microvesicles are typically larger than exosomes, we speculate that in addition to release of exosomes by the fusion of multivesicular bodies with the plasma membrane^29^, neurons likely shed microvesicles from the plasma membrane. Microvesicles are important participants in cell–cell communication by mediating horizontal transfer of macromolecules between neighboring cells^30^. The role of microvesicles versus exosomes released from neurons is not well characterized.

Neuronal EVs may be more homogenous in morphology compared to those released from other cell types^11^. We found that EVs released by neurons are largely round with few detectable vesicles exhibiting irregular phenotypes such as tubular vesicles or containing filamentous structures. Like EVs from other cell types, we observed vesicles containing internal vesicles and EVs contacting each other. The later of these phenotypes has been attributed to high centrifugal forces used to concentrate vesicles from solution^15^; however, we also observe these phenotypes in vesicles concentrated by ultrafiltration where high g-forces are not used.

The discovery of macromolecular clustering on the luminal face of EV membranes is a potentially important phenomenon for EV physiology by acting as a functional microdomain. However, to begin to understand the function of these structures, several questions remain. First, are these microdomains specific to an EV subtype? Since we observed clusters more frequently on medium-sized EVs, we speculate that this phenotype may be associated with microvesicles as opposed to exosomes, which are typically smaller.

Determining when these clusters form might shed insight into their function. We anticipate three scenarios of when these clusters might form: (1) at the time of EV formation (2) extracellularly to facilitate docking and fusion or (3) under pathological conditions that favor macromolecular aggregation. Microvesicle formation involves recruitment of lipids, RNAs and proteins to the plasma membrane resembling that of viral particle formation^31^. If assembly of this platform drives EV budding, then determining the molecular composition within the cluster could reveal the machinery used to generate EVs. This clustering might also serve to recruit cargo into the vesicle for selective packaging.

If the clusters assemble extracellularly, they could function in the communication of the EV with recipient cells. Clustering does not appear to extend to the extracellular face of the membrane, so it is unclear whether this clustering would affect transmembrane receptors. However, it could affect membrane lipids that would allow for recognition by the recipient cell^32^. The clusters frequently occur where membranes bulge outward, suggesting that they may induce membrane curvature that could be important for recognition by recipient cells. If protein clustering generates a signaling platform, then proteins or lipids within this platform could determine cell-type specific recognition of EVs^33^.

Lastly, if clusters form under conditions that favor aggregation, they may have the potential to disrupt normal EV-cellular function. In fact, EVs transmit pathological proteins from one cell to another in the brain. For instance, EVs transfer alpha-synuclein and hyper-phosphorylated tau in Parkinson’s disease and Alzheimer’s disease models, respectively^34^. They may contribute to the spread of pathogenic proteins in the brain during neurodegenerative disease. Alternatively, they may help neurons transport macromolecules (such as lipids) damaged by oxidative stress to glial cells for support^35^. An important future direction will be to identify the composition of molecules in the clusters and whether pathogenic proteins susceptible to aggregation are present. Recent advances in cryo-correlative light and electron microscopy offer a promising strategy to address this outstanding question. Given the involvement of EVs in pathologies such as neurodegenerative disease, the proteins within these clusters may be useful targets for treating disease.

In summary, we performed a detailed characterization of neuronal EVs by cryoEM and discovered macromolecular clusters on the luminal membrane. We speculate that these clusters constitute a functional microdomain within vesicles and play an important role in EV physiology.

## Materials and methods

### Primary culture of hippocampal neurons

All animal work was approved by and performed in accordance with the Institutional Animal Care and Use Committee at Janelia Research Campus (IACUC 16–146) and the Canadian Council of Animal Care at the University of Alberta (AUP#3,358). Sprague–Dawley timed pregnant rats were purchased from Charles River Laboratories and housed in our facility for one week prior to birth. Primary cortical neurons were prepared as previously described^35^. Briefly, the cortices were dissected from P0 Sprague–Dawley rat pups, digested with papain (Worthington Biochemical), triturated, and filtered with a cell strainer. Neurons were grown for 10 days on poly-d-lysine coated 10-cm plastic culture plates in serum-free NbActiv4 medium (BrainBits) containing antibiotic–antimycotic (Gibco) at 37 °C. 2 µM cytosine-beta-d-arabinofuranose (AraC, ThermoFisher Scientific) was added on DIV 2. Half the media was replaced every 3 days with fresh NbActiv4 without AraC.

### Fluorescence microscopy

Neurons were plated on poly-d-lysine coated glass coverslips. After 10 days in culture neurons were washed 3 times in PBS, fixed in 4% paraformaldehyde, blocked with PBS containing 1% BSA plus 0.02% TritonX-100, immunostained with mouse monoclonal anti-β3-tubulin (Biolegend, 801201, RRID:AB_2313773) and goat anti-mouse alexafluor488 (Invitrogen, A11001) and stained with DAPI (Abcam, ab228549). Neurons were routinely monitored for viability and mycoplasma contamination by imaging nuclei stained with DAPI. Imaging was performed using a Laser Scanning Confocal Microscope (LSM880, Zeiss) equipped with a plan-apochromat 63 × oil objective (Zeiss, NA = 1.4) and ZEN software (Zeiss).

### Purification of extracellular vesicles

Media of DIV 9 neurons was reduced to 5 mL and 16 h later, neuron-conditioned medium was collected. EV Centrifugation: Neuron-conditioned medium from a 10-cm plate was centrifuged at 2,000×g at 4 °C for 10 min to remove dead cells or cell debris followed by centrifugation at 10,000×g at 4 °C for 20 min. The supernatant containing EVs was centrifuged at 300,000×g at 4 °C for 3 h in a TLA-110 fixed angle rotor (k-factor 13) (Beckman Coulter). The pellet containing EVs was resuspended in 10 µL PBS. EV Ultrafiltration: media from three 10-cm plates was pooled and centrifuged at 2,000×g at 4 °C for 10 min, then added to the top chamber of an Amicon Ultra centrifugal filter unit with a 100 kDa molecular weight cutoff (Millipore-Sigma) and centrifuged at 4,000×g at 4 °C for 20 min until final volume reached approximately 200 µL.

### Western blotting

Neurons on 10-cm plates were washed 3 times in PBS and lysed in lysis buffer (20 mM Tris pH 7.4, 100 mM NaCl, 5 mM EDTA, 1% Triton-X 100, supplemented with 1 × HALT protease and phosphatase inhibitor cocktail (ThermoScientific, 78,440). Following centrifugation of EVs from neuron-conditioned media, pellets were resuspended in 25 µL lysis buffer. SDS-PAGE sample loading buffer was added to samples which were subsequently resolved by SDS-PAGE and processed for Western blotting. Primary antibodies used include rabbit polyclonal anti-syntenin (Abcam, ab19903, RRID:AB_445200, 1:1,000), mouse monoclonal anti-flotillin-1 (BD Biosciences, 610,821, RRID:AB_398140, 1:1,000), mouse monoclonal anti-gp96 (R&D systems, MAB7606, clone 816,803, 1:500), and mouse monoclonal anti-β3-tubulin (Biolegend, 801,201, RRID:AB_2313773, 1:5,000). HRP-conjugated affinity purified secondary anti-rabbit and anti-mouse were purchased from Jackson Immunoresearch Labs.

### Transmission electron microscopy

For negative staining, 5 µL drops of EV suspension were adsorbed onto glow-discharged copper grids coated with Formvar-carbon and stained with 1% uranyl acetate in water. Samples were imaged in a Tecnai Spirit electron microscope (FEI, Hillsboro, OR) operating at 80 kV equipped with an Ultrascan 4,000 digital camera (Gatan, Inc., CA).

### Cryo-electron microscopy

For cryoEM, 3 µL of fresh EV suspension were adsorbed onto glow-discharged Quantifoil R1.2/1.3 or R2/1 400 mesh copper grids as well as 2 nm carbon layer coated Quantifoil R1.2/1.3 400 mesh gold grids, blotted either for 3 s at 4 °C and targeted relative humidity level of 100% and plunge frozen using a Vitrobot Mark IV (Thermo Fisher Scientific, MA) or for 4–6 s at 4 °C and ~ 86% and plunge frozen using a Leica EM GP (Leica Microsystems Inc, Buffalo Grove, IL). The samples were imaged in an FEI Tecnai F20 TEM operated at 200 kV through a Gatan 626 side-entry cryo holder. This F20 TEM is equipped with a standard Field Emission Gun (s-FEG) and a K2 Summit camera (Gatan, Inc., CA). Images were recorded with a pixel size of 1.223 or 1.478 Å/px at a dose rate of ~ 8 e^−^/px/s with a 3,708 × 3,838-pixel field of view. A total electron dose of ~ 60–80 e^−^/Å^2^ using dose fractionation, while 0.2 s frames were aligned on the fly using a default algorithm in SerialEM. Images were low pass filtered for enhanced contrast (Gaussian cutoff abs = 0.125).

### 3D tomography acquisition

Dose-symmetric tilt-series were acquired on a Titan Krios TEM (HHMI, Janelia Krios2, Thermo Fisher Scientific, MA) operated at 300 kV equipped with a high-brightness Field Emission Gun (x-FEG), a Bioquantum energy filter and a K3 camera (Gatan, Inc., CA). Tilt-series were acquired automatically using SerialEM using a dose-symmetric tilt-scheme starting from 0° with 3° step increments to + 3°, + 6°, then − 3°, − 6°, etc. until − 48°^36^. The accumulated dose over the entire tilt series was ~ 82 e^−^/Å^2^ with a dose rate of 15 e^−^/px/s. 10 movie frames per tilt with ~ 0.248 e^−^/Å^2^ per frame were collected using dose fractionation mode. Images were recorded at a nominal defocus of − 3 µm and at a nominal magnification of 42,000 × in super-resolution mode. The calibrated pixel size is 2.2 Å/px (1.1 Å/px super-resolution) corresponding to a calibrated magnification of 22,727x. Movie frames were aligned and CTF was estimated using cisTEM^37^. Tilt series alignment and tomographic reconstruction were done using the IMOD package^38,39^. Segmentation was done with Amira (Thermo Fisher Scientific, MA).

### Macromolecular bubbling assay

Images were recorded on the same Titan Krios that was used for tomography (see above). Multiple 60 frame-images were recorded at a dose rate of ~ 15 e^−^/px/s and pixel size of 1.07 Å/px using super-resolution counting mode (0.535 Å/px), accumulating ~ 60 e^−^/Å^2^ per image. A defocus of − 3.5 µm was applied.

### Analysis of extracellular vesicles

EVs were identified by the presence of a bilayer membrane. EVs and clusters were manually traced, and the area, diameter, and roundness were calculated using ImageJ software. For roundness, only vesicles entirely within the field of view were quantified. Vesicles more than 20% out of the field of view were excluded from quantification of vesicle diameter. Statistical analysis was performed using SPSS Statistics 17.0 (IBM) or GraphPad Prism 8. Student’s *t*-test was used for comparison of groups. Since no difference (*t*-test, *p* = 0.743) was detected in dUC EV diameter from cells stimulated with 10 µM NMDA for 18 h (n = 32) versus unstimulated cells (n = 227), this data was combined in the histograms showing distribution of diameters.

### Reporting

We have submitted all relevant data of our experiments to the EV-TRACK knowledgebase (EV-TRACK ID: EV200012)^40^.

## Supplementary information

Supplemental Figure 1.

Movie S1.

Movie S2.
