# Supplementary material for: Microdomains form on the luminal face of neuronal extracellular vesicle membranes
Source: Sci Rep. 2020 Jul 20;10:11953. doi: 10.1038/s41598-020-68436-x (PMC7371872; doi:10.1038/s41598-020-68436-x)
Supplement: Supplementary file 1 — Supplemental Figure 1. [file 41598_2020_68436_MOESM1_ESM.pdf]

SUPPLEMENTAL MOVIES

Movie S1. Aligned tilt series of one of the EVs with a dense macromolecular cluster near the membrane which was used to generate a tomogram used for segmentation as shown in Figure 2C.

Movie S2. Aligned tilt series of EVs used for segmentation in Figure 4F.

SUPPLEMENTAL FIGURE

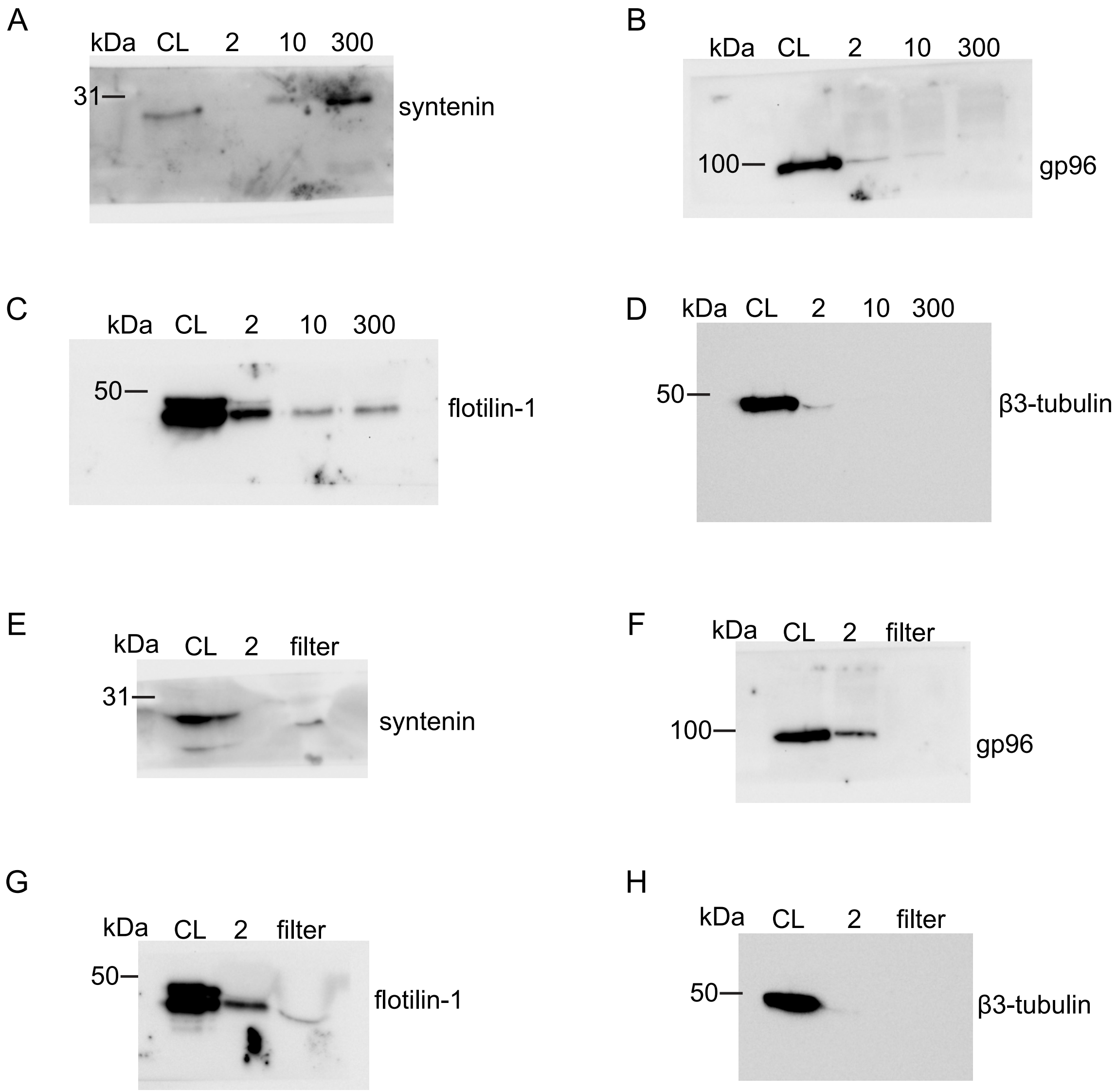

Supplementary Figure 1. Original unprocessed Western blots. (A-D) dUC fractions corresponding to Figure 1F. (E-H) UF fractions corresponding to Figure 1I. CL, cell lysate.
